# The Dark Triad of Personality in the Context of Health Behaviors: Ally or Enemy?

**DOI:** 10.3390/ijerph18084113

**Published:** 2021-04-13

**Authors:** Małgorzata Dębska, Paweł Dębski, Jacek Polechoński, Michał Rozpara, Rajmund Tomik

**Affiliations:** 1 Institute of Sport Sciences, The Jerzy Kukuczka Academy of Physical Education in Katowice, Mikołowska 72A, 40-065 Katowice, Poland; m.debska@awf.katowice.pl (M.D.); m.rozpara@awf.katowice.pl (M.R.); r.tomik@awf.katowice.pl (R.T.); 2 Chair and Clinical Department of Psychiatry, Faculty of Medical Sciences in Zabrze, Medical University of Silesia in Katowice, Poniatowskiego 15, 40-055 Katowice, Poland; pdebski@sum.edu.pl

**Keywords:** Health Behavior Inventory, healthy lifestyle, socially aversive personality traits, narcissism, Machiavellianism, psychopathy

## Abstract

The main aim of this manuscript was to present a preliminary verification of the relations between the Dark Triad of personality traits and health-oriented behaviors in university students. The study involved 143 healthy students (94 female and 49 male) from the Silesian Voivodeship (Poland). The diagnosis of the elements of the Dark Triad of personality was carried out using a psychological examination based on the following tests: TriPM-41, Mach IV, NPI. The intensity of the health behaviors was examined using the Health Behavior Inventory developed by Z. Juczyński. The Health Behavior Inventory is positively correlated with narcissism and its self-sufficiency component and negatively with psychopathic disinhibition. For the Health Behavior Inventory’s subscales, a positive relationship was observed between Positive Mental Attitude and narcissism, most of its components, and psychopathic boldness. The analysis of correlations with the division of the subjects into the Dark Triad traits ≤Me and >Me revealed that a significant positive correlation between the general intensity of the health behaviors and the intensity of narcissism mainly concerns university students with a lower level of this trait and its components. Therefore, it can be presumed that narcissism, although considered as a socially aversive trait, is associated with pro-health behaviors.

## 1. Introduction

The high incidence of chronic noncommunicable diseases (NCDs) has been a major challenge for public health actors for many years. According to many epidemiological studies, the overriding cause of this problem is unhealthy lifestyle [1]. Therefore, a very important objective of the research is to identify the determinants of health behaviors [2].

The diagnosis of the degree of implementation of individual health behaviors is a relatively popular topic of scientific publications [3]. However, most of the studies on this subject so far have focused on single health behavior. The verification of the intensity of several health practices is less frequent [4,5], although it is essential for the assessment of the lifestyles of the populations studied. Given the above, questionnaires such as Health Behavior Inventory were developed, which aimed to collect general information on the intensity of health behaviors and their basic categories [6].

A variety of factors (determinants) that favor caring for health (manifested in health behaviors) have been verified in the health sciences [7,8]. The identification of the effect of these determinants on undertaking or failure to undertake health-oriented activities is a key aspect of the effectiveness of health promotion and prevention [9]. This is because it enables the development and improvement of theoretical models of modification of behaviors into health behaviors and the development of effective programs to promote a healthy lifestyle [10]. In this context, the diagnosis of factors of a psychological nature, considered as determinants of the general willingness to undertake or abandon various behaviors, seems to be an important research area. Personality traits are especially critical here, as it is the personality that characterizes the main behavioral dispositions that manifest themselves in a situational context, making it one of the main factors predisposing human to undertake specific activities to maintain or improve health. Most publications dealing with the abovementioned problems focus on psychological properties which undoubtedly determine the health potential of a person, i.e., psychological determinants of health behaviors (self-esteem, self-efficacy, optimism, sense of coherence, or mental resilience) [11,12,13,14]. Few studies have verified the relationship between health care and properties often considered undesirable from the perspective of personal and social functioning, such as socially aversive traits [15,16,17].

In the beginning, the interest of researchers in antisocial personality traits was attracted to the clinical and criminological aspects, reflected in the clinical and forensic psychology research. Over time, it was noticed that these traits not only indicate the pathology of psychological function but, according to the concept of personality traits, are common and occur in people with different intensity [18]. The evolution of the understanding of antisocial tendencies in behavior from clinical and criminological issues towards personality problems resulted in the development of research on these phenomena in the field of psychology of personality. In the course of this research, a construct combining antisocial elements of the subclinical nature such as narcissism, Machiavellianism and psychopathy was developed, forming, for the purposes of their description, a category termed the Dark Triad (DT) [18]. This concept includes three personality traits: narcissism (defined by grandiosity, entitlement, dominance, and superiority), Machiavellianism (defined by manipulation, self-service, amorality and deceit), and psychopathy (defined by impulsivity and thrill-seeking along with low empathy and anxiety). The above features reflect the tendencies for socially aversive actions, whereas their high intensity characterizes people who put their good and success before the public interest [18,19,20,21]. DT is a construct that has been widely discussed in many psychological subdisciplines that explore both negative and positive aspects of human behavior. 

In the last two decades, DT has been a particularly popular research problem among the representatives of social psychology, work psychology, and organizational psychology. The results of the research indicate that the elements of this construct can support individual success as they are connected with self-interest and the need for self-realization [22]. Narcissistic vanity, the need for admiration from others and a sense of self-importance appear to predispose people to care for their health, especially in the physical dimension through personal hygiene, physical activity, and rational diets [18]. Furthermore, the Machiavellian determination to achieve personal goals [23] can favor regular health behaviors, which are critical to their healthy character. 

Few publications to date on the relationship between socially aversive traits and caring for health show significant relationships [15,16,24,25,26]. Most of them are aimed at the relation of DT and undertaking risky behaviors. The link between healthy lifestyle and the Dark Triad seem to be the results of investigations conducted by Gerber et al., Onley et al., and Bahmani et al. [16,27,28]. They showed that some physical health behaviors depend on the subjects’ level of mental toughness (MT), defined as “a personal capacity to produce consistently high levels of subjective (e.g., personal goals or strivings) or objective performance (e.g., sales, race time, grade per average) despite everyday challenges and stressors as well as significant adversities” [29]. Gerber et al. observed that physical activity in subjects with high MT was more often at the level recommended for health as compared to those with lower MT [27]. At the same time, it was observed that MT, commonly associated with psychological well-being, is also positively correlated with the Dark Triad [16]. Furthermore, the positive relationship of the volume of PA with MT and DT in adults was also confirmed [17,30]. This leads to the presumption that individuals with high levels of DT traits might be more likely to pursue behaviors requiring regularity, organization, engagement or renouncing, like those of a health nature [17]. At the same time, the abovementioned publications have explained the relationship between DT and PA with the mediating effect of MT, indicating the need for further explorations in this field [30]. Therefore, it can be expected that there are relationships between the DT traits and the intensity of different health behaviors [27,30] whose strength and direction are still poorly recognized. 

Therefore, the main aim of this manuscript was to present a preliminary verification of the relations between socially aversive personality traits (the Dark Triad of personality) and health-oriented behaviors in university students.

## 2. Materials and Methods

### 2.1. Study Group

The study involved 143 healthy students (94 female and 49 male, mean age 23.7 ± 0.9 years, mean BMI 23.8 ± 2.4 kg/m^2^) of the last year of Master’s degree programs aimed at educating future health promoters (tourism and recreation, physical activity and nutrition, physical education) in the Silesian Voivodeship (southern Poland). This group is expected to be characterized by a high level of interest in and knowledge of healthy lifestyles and a positive attitude towards caring for health. 

### 2.2. Measures

The diagnosis of the elements of DT traits was carried out using psychological tests. The following psychometric tests were used:

1. The Polish adaptation of Triarchic Psychopathy Measure (TriPM-41) to measure psychopathy (TRiMP: Patrick; Polish version: Pilch et al.) [31,32]. The questionnaire is based on a triarchic approach, which includes boldness (striving for dominance, courage, resistance to stress), disinhibition (tendency to be impulsive) and meanness (the tendency for being cruel and aggressive). The test consists of 41 statements to which the examined person responds in a 4-point response rate: 1–“true”, 2–“somewhat true”, 3–“somewhat false”, 4–“false”. The overall score for psychopathy is obtained by adding up the points indicated for all test items. The scoring of some answers is reversed. TriPM-41 demonstrated good psychometric properties in the general population [32]. For the current study, estimated reliability for TRiMP-41 is 0.94 and from 0.72 to 0.84 for individual subscales.
2. The Polish version of the Mach IV scale by Christie and Geist [33,34] to measure Machiavellianism, i.e., the tendency to manipulate others and treat them unemotionally. The questionnaire consists of 20 items. The participant responds to the test item on a seven-point scale, in which 1 means “I completely disagree” and 7 “I completely agree”. Some test items have reversed scores. The sum of the points obtained from all the respondents’ answers constitutes the overall Machiavellian score The questionnaire demonstrates satisfactory reliability in general population [32]. For the current study, the estimated reliability for Mach IV is 0.70.
3. The Polish adaptation of Narcissistic Personality Inventory (NPI: Raskin and Hall; Polish version: Bazińska and Drat-Ruszczak) [21,35] to examine narcissism. This questionnaire consists of four subscales: the need for admiration (demanding to be noticed), vanity (self-approval), self-sufficiency (confidence in one’s competence) and leadership (confidence in high influence on others). It contains 34 affirmative items, to which the participant responds on a five-point scale, where 1 means “It is not me” and 5 means “It is me”. The estimated reliability for NPI in the current study is 0.94 and from 0.70 to 0.90 for individual subscales.

The intensity of health behaviors was examined using the Health Behavior Inventory developed for adults by Z. Juczyński [6]. This questionnaire is the only Polish tool for measuring the overall intensity of health practices. This is a psychometric test, composed of 24 statements describing health behaviors in adults, both healthy and ill. For each item of the questionnaire, the frequency of the indicated behavior is assessed on a five-point scale (1–5), where 1 means “almost never” and 5 means “almost always”. The intensity of the health-promoting activities is verified in general terms (HBI) and within the four main categories: Proper Eating Habits (PEH) (type and frequency of food consumed), Preventive Actions (PA), (following health recommendations, obtaining information about health and illness), Prohealth Activities (PhA) (everyday habits: sleep, recreation, physical activity), Positive Mental Attitude (PMA) (avoiding strong emotions, tensions, and stressful and depressing situations). The tool reliability for the current study is 0.81 for the entire inventory, whereas for individual subscales, it ranges from 0.60 to 0.80.

### 2.3. Procedures

The research was conducted in the laboratories of the Academy of Physical Education in Katowice in an auditorium form. Questionnaires were filled in in groups of up to 15 people to provide students with quiet during the test. The people who expressed their willingness to participate in the study were assigned to specific editions of the test and informed about the date by email. Before each test, the survey supervisors informed participants about the aim of the study and discussed the filling instructions. 

### 2.4. Ethics

The study procedures were reviewed by the Research Ethics Committee of the Medical University of Silesia in Katowice (PCN/0022/KB/277/19) and they were in the accordance with the Helsinki Declaration. All subjects were familiarized with the aim of the study prior to data collection. Participants took part in the research voluntarily and could discontinue their participation at any time. They provided written consent for the use of information collected during the examination.

### 2.5. Statistical Analyses

Statistica 13.0 (StatSoft, Inc., Tulsa, OK, USA) was used to carry out statistical calculations. The normality of data distribution was assessed with the Shapiro–Wilk test. The statistical significance of the differences in HBI and DT traits between sexes was determined by Student’s t-test and Mann–Whitney’s U test. Next, a series of Pearson’s correlations were computed to calculate the associations between HBI, its categories and DT traits. Furthermore, the partial correlations were conducted, in order to check the potential spurious associations between HBI, its categories and DT traits in the study group.

## 3. Results

The general index of intensity of health behaviors (HBI) in the university students was at a mean level, with 5th sten in women (81.69 ± 12.11 points) and 6th sten in men (81.43 ± 12.40 points). The highest level of health-related activities was observed in Preventive Actions subscale and the lowest in Prohealth Activities subscale. Neither general nor subscale HBI results were significantly differentiated depending on sex. However, it was observed that men were involved in Prohealth Activities slightly more often in the two first subscales (Positive Mental Attitude, Preventive Actions) and slightly more rarely in twosubscales (Proper Eating Habits, Prohealth Activities) than women (Table 1).

The average intensity of narcissism of the university students who participated in the study was significantly higher in men compared to women (*p* < 0.01). This differentiation concerned narcissistic vanity as a higher level characteristic of male study participants in the study group. No significant sex differences were observed for the intensity of the other DT traits (Table 1). 

HBI was significantly positively correlated with narcissism (0.18) and the self-sufficiency component (0.28) and negatively with psychopathic disinhibition (−0.27). Correlation analysis at the level of HBI subscales revealed a significant positive relationship of Positive Mental Attitude results with narcissism (0.25) and most of its components and with psychopathic boldness (0.19). Positive Mental Attitude was also significantly negatively correlated with psychopathic disinhibition (−0.23). Furthermore, a significant positive correlation between activities related to Preventive Actions and meanness was observed (0.19) (Table 2).

The partial correlations results revealed that the association between health behaviors intensity and narcissism when controlling the effect of Machiavellianism and psychopathy was spurious in examined students. It was observed that the positive correlations between Positive Mental Attitude and the overall narcissism and narcissistic vanity were stronger (respectively 0.21, 0.28) compared with those indicated in the zero-order correlations. At the same time, the significant increases in PMA activities along with the intensification of self-sufficiency and leadership were recognized as spurious. After the elimination of the influence of relations between HBI and other components of narcissism, a significant positive relationship between narcissistic self-sufficiency and Preventive Actions (0.16) as well as Proper Eating Habits (0.16) was revealed. Whilst controlling for the effects of narcissism and psychopathy, the significant negative correlation between HBI and Machiavellianism was observed (−0.18). The negative correlation between Machiavellianism and HBI was revealed in particular in the Positive Mental Attitude (0.17) and Proper Eating Habits (0.17) categories. The partial correlation results presented a significant positive dependence of HBI on the intensity of psychopathic boldness (0.16), in particular PMA activities (0.25). After controlling for the effects of boldness and meanness, significant negative correlations between psychopathic disinhibition with the majority of health behaviors categories were revealed (Table 2).

## 4. Discussion

In the last decade, antisocial personality traits started to be assessed in the context of their relationship with health in general. In the few studies concerning the abovementioned problems, apart from positive relations of DT components with anti-health behaviors [36,37,38], authors have shown important relationships of the DT traits with the element of the mental toughness [16] and also with physical activity as one of the most important health behaviors today [17,30]. Given the above, the present study aimed to initially verify the relationship between the DT traits and the intensity of health behaviors in students of majors related to health promotion (tourism and recreation, physical activity and nutrition, physical education). It is worth emphasizing that this is the first publication to assess the relationship between DT and caring for health expressed as the involvement in health behaviors. 

The results of our research showed that the general intensity of health behaviors in the students participating in the study was on a mean level (5th sten in women, 6th sten in men) even though they were students in the last year of university were preparing to performing the roles of health promoters in the future. The mean HBI value (total: 81.60 ± 12.17 women: 81.69 ± 12.11; men: 81.43 ± 12.40) was slightly higher than that obtained by students in population studies (80.62 ± 15.34) [6]. Contrary to the abovementioned study, no sex differences in caring for health were found in the students surveyed in our study. The diagnosed students were characterized by the highest intensity of health behaviors in terms of Preventive Actions (3.88 ± 0.85), whereas the lowest intensity was found for Prohealth Activities (2.69 ± 0.57) [6]. This leads to the conclusion that the students in our study followed the doctor’s recommendations, participated in preventive medical check-ups, and were willing to learn about health and illness, while they reported less frequent daily healthy habits in their lives (the recommended dose of sleep, maintaining a proper posture, regular physical activity). 

A positive correlation was demonstrated between HBI and narcissism. The deepened analysis of this relationship revealed that it is significant only for one of its categories—the Positive Mental Attitude. The relationship between narcissism and human mental potential has also been confirmed by the studies of other authors [16,17,38,39], whose results demonstrated, among others, a significant positive correlation between narcissism and self-efficacy or self-esteem. The high self-esteem and belief in the abilities resulting from these properties provide the basis for taking a specific action and imply the necessary effort [21]. In the context of health behaviors, they can translate into commitment and perseverance, which, in the case of this type of activity, is essential for the health benefits that arise through regular and long-term action. 

Positive correlations between narcissism and PMA are consistent with the relationships demonstrated in the literature between this trait and beneficial psychological characteristics (the healthy narcissism trend) [40,41], which, in the context of our research, is exemplified by avoiding excessively strong emotions, stress, tensions or depression. Narcissistic self-sufficiency and vanity are associated with the belief in one’s competence and resources and the belief in the ability to lead others [40]. Such beliefs allow the person to build a positive self-image and can be linked to the feeling of satisfaction with life [21]. It should also be stressed that narcissists usually want to see their lives and activities as successful, well-planned, competent, so that they may tend to protectively reinforce their self-esteem. Therefore, the increase in PMA with rise of narcissism shown in this study could also be due to the narcissistic protection of self-esteem. This relationship may also result from the highly flattering, although not necessarily true, cognitive patterns. A strong conviction of one’s competence or leadership skills and a belief that one deserves admiration (also due to the attractiveness of his or her own body or health) should imply a positive mental attitude. It is hard to imagine that people who are vain and convinced of their high level of competence would at the same time tend to show negative emotions, stress and depression.

Furthermore, the results of our study revealed the significant correlation between narcissistic vanity and PMA. It may be translated as willingness to implement health behaviors (e.g., healthy diets and physical activity) to ensure an attractive appearance. This is consistent with the observation made by Back et al. [39].

According to the results, the significant negative correlation between HBI and Machiavellianism was observed. High scores in Machiavellianism encourage antisocial attitudes and tendencies which might manifest in engaging in different negative health behaviors. Machiavellianism is traditionally associated with deficits in empathy, which is related to the objective approach to people and manifests itself in the tendency to manipulate them. In the light of the literature, it seems that Machiavellians, just like they do not care about relationships with other people, are also characterized by neglecting their own health. Apart from antisocial behavior, Machiavellianism is associated with anti-health behaviors such as internet addiction [42,43]. It is also suggested that Machiavellianism is associated with deteriorating mental health (positive associations with depression, paranoia, alexithymia), as well as with low self-esteem and a weakened task-oriented coping style [44]. People with Machiavellianism and psychopathy may be predisposed to using alcohol more often and smoking cigarettes, as well as being less willing to eat breakfast, exercise regularly or visit doctors [26].

The results of our research showed negative relations between psychopathic disinhibition, HBI in general and the majority of health behaviors categories. These relationships were as expected due to the nature of this trait, which is associated with impulsivity, difficulty in planning, difficulty in predicting the consequences, problems with self-control, or poor ability to delay gratification [31]. The abovementioned features do not favor the consistency of actions which is crucial in health behaviors. According to the results of other researchers, psychopathy is positively correlated with antisocial, risky behaviors [24,25].

In our study, we also observed the significant relationships between psychopathic boldness, overall HBI and the positive mental attitude activities. It is worth pointing out that boldness is connected with self-confidence, courage, and the search for impressions and resistance to stress. Therefore, psychopathic boldness may encourage people to engage in physical activity, for example, because such health practices may become an opportunity to seek new experiences and sensations. Physical activity and recreation is in this case in line with the personality-related boldness. 

It should be emphasized that the present study aimed to initially identify the relationships between socially aversive traits and health behaviors and therefore it should be viewed in the context of several limitations. A tool for the general assessment of the intensity of health behaviors using only the diagnosis of one parameter (frequency of health behaviors) was used. Further research should involve the use of tools for a detailed multiparameter diagnosis of individual behaviors (e.g., accelerometers, standardized dietary interviews), which will enable their assessment in relation to the recommendations developed by international public health organizations (American College of Sports Medicine, European Food Safety Authority, World Health Organization) and consequently a thorough verification of their relationship with DT traits. We are aware that the results of this study do not provide a complete answer to the question asked in the title. The answer requires further verification of this problem, e.g., by extending the research to include various population groups (including diverse age, health behaviors and health literacy, etc.).

## 5. Conclusions

The results of the study indicated a positive correlation between the intensity of health behaviors, narcissistic vanity and self-sufficiency in the surveyed students. This relationship mainly concerned activities related to positive mental attitudes. Furthermore, it was observed that the increase in health behaviors is related to the rise of psychopathic boldness. Additionally, the Health Behavior Inventory was significantly negatively related to Machiavellianism and psychopathic disinhibition. Therefore, it can be presumed that some Dark Triad traits, although considered to be antisocial, can be associated with positive health behaviors.

## Figures and Tables

**Table 1:** The Dark Triad traits and Health Behavior Inventory results.

Characteristics	Total (*n* = 143) [Points]	Women (*n* = 94) [Points]	Men (*n* = 49) [Points]	U
Health Behavior Inventory	81.60 ± 12.17	81.69 ± 12.11	81.43 ± 12.40	ns
Positive Mental Attitude	3.51 ± 0.71	3.47 ± 0.74	3.57 ± 0.65	ns
Preventive Actions	3.88 ± 0.85	3.87 ± 0.82	3.89 ± 0.90	ns
Proper Eating Habits	3.52 ± 0.73	3.56 ± 0.69	3.45 ± 0.82	ns
Prohealth Activities	2.69 ± 0.57	2.70 ± 0.61	2.68 ± 0.50	ns
Narcissism	104.27 ± 22.68	101.74 ± 21.67	109.08 ± 24	*
Demanding admiration	31.99 ± 9.65	31.12 ± 8.31	33.67 ± 11.72	ns
Vanity	15.85 ± 4.69	15.06 ± 4.26	17.35 ± 4.89	**
Self-sufficiency	24.16 ± 4.87	23.84 ± 5.01	24.78 ± 4.58	ns
Leadership	32.37 ± 8.29	31.76 ± 8.22	33.55 ± 8.39	ns
Machiavellianism	79.73 ± 13.32	78.54 ± 13.52	82 ± 12.76	ns
Psychopathy	47.88 ± 14.05	47.56 ± 14.36	48.49 ± 13.56	ns
Boldness	22.63 ± 6.10	21.99 ± 5.89	23.86 ± 6.35	ns
Disinhibition	14.65 ± 8.18	15.20 ± 8.33	13.59 ± 7.85	ns
Meanness	10.69 ± 5.23	10.50 ± 5.25	11.04 ± 5.22	ns

Notes: comparison is significant at the * 0.01, ** 0.001.

**Table 2:** Intensity of health behaviors vs. the Dark Triad traits and their components (zero-order and partial correlations).

Independent Variables	Pearson Correlations	Dependent Variables				
		Health Behavior Inventory	Positive Mental Attitude	Preventive Actions	Proper Eating Habits	Prohealth Activities
Narcissism (1)	Zero-order	0.180 *	0.246 *	0.029	0.015	−0.069
	Partial (2–3)	0.130	0.280 ***	0.029	0.075	−0.032
Demanding admiration (A)	Zero-order	−0.009	0.155	−0.038	−0.070	−0.104
	Partial (B-D)	−0.082	−0.037	−0.093	0.004	−0.105
Vanity (B)	Zero-order	0.074	0.202 **	0.037	−0.066	0.037
	Partial (A, C-D)	0.096	0.212 *	0.022	−0.041	0.032
Self-sufficiency (C)	Zero-order	0.277 *	0.239 *	0.164	0.161	0.040
	Partial (A-B, D)	0.160 *	0.075	0.160 *	0.156 *	0.041
Leadership (D)	Zero-order	0.105	0.217 *	0.018	0.041	−0.084
	Partial (A-C)	−0.013	0.065	−0.051	−0.017	−0.031
Machiavellianism (2)	Zero-order	−0.134	−0.071	−0.031	−0.101	−0.117
	Partial (1, 3)	−0.182 *	−0.169 *	−0.073	−0.167 *	−0.114
Psychopathy (3)	Zero-order	−0.098	−0.083	−0.006	−0.052	−0.078
	Partial (1–2)	0.007	−0.081	0.017	−0.007	0.104
Boldness (E)	Zero-order	0.109	0.192 *	0.040	0.096	0.002
	Partial (F-G)	0.156 *	0.254 **	−0.006	0.100	0.112
Disinhibition (F)	Zero-order	−0.267 *	−0.234 *	−0.146	−0.142	−0.157
	Partial (E, G)	−0.272 **	−0.258 **	−0.221 **	−0.171 *	−0.100
Meanness (G)	Zero-order	0.058	−0.031	0.186 *	0.019	0.007
	Partial (E-F)	0.180	0.037	0.249	0.071	0.113

Notes: 1–narcissism as a covariate, 2–Machiavellianism as a covariate, 3–psychopathy as a covariate, A–demanding admiration as a covariate, B–vanity as a covariate, C–self-sufficiency as a covariate, D–leadership as a covariate, E–boldness as a covariate, F–Disinhibition as a covariate, G–meanness as a covariate, correlation is significant at the * 0.05; ** 0.01, *** 0.001; level (1-tailed).

## Data Availability

The data presented in this study are available on request from the corresponding author.
